# Early versus Delayed Phacoemulsification and Intraocular Lens Implantation for Acute Primary Angle-Closure

**DOI:** 10.1155/2020/8319570

**Published:** 2020-03-24

**Authors:** Yun-Hsuan Lin, Cheng-Hsiu Wu, Shih-Ming Huang, Chen Hsieh, Henry Shen-Lih Chen, Wan-Chen Ku, Ming-Hui Sun, Wei-Wen Su

**Affiliations:** ^1^Department of Ophthalmology, Chang Gung Memorial Hospital, Keelung Branch, Taiwan; ^2^Chang Gung University College of Medicine, Taoyuan, Taiwan; ^3^Department of Electro-Optical Engineering, National Taipei University of Technology, Taipei, Taiwan; ^4^Department of Radiation Oncology, Chang Gung Memorial Hospital, Keelung Branch, Taiwan; ^5^Department of Radiation Oncology, Chang Gung Memorial Hospital, Linkou Branch, Taoyuan, Taiwan; ^6^Department of Ophthalmology, Chang Gung Memorial Hospital, Linkou Branch, Taoyuan, Taiwan

## Abstract

**Purpose:**

To compare the effects of early phacoemulsification and intraocular lens implantation (phaco/IOL), delayed phaco/IOL after initial laser peripheral iridotomy (LPI), and conventional LPI alone in patients with acute primary angle-closure (PAC).

**Methods:**

Patients with acute PAC were included in the study, and those with secondary glaucoma, prior ocular trauma, or other ocular diseases and those who had undergone ocular surgeries previously were excluded. Patients were categorized into three groups: Group A, which underwent primary phaco/IOL after acute PAC; Group B, which underwent LPI initially after acute PAC, followed by phaco/IOL within 6 months; and Group C, which underwent LPI alone. The IOP control success at 12 months as well as changes in ocular characteristics and the number of antiglaucoma medications used after the treatment among the groups were evaluated.

**Results:**

Eighty-one eyes were included in the study: 24 eyes in Group A, 23 eyes in Group B, and 34 eyes in Group C. The linear mixed model analysis demonstrated considerable IOP control in Groups A and B. Visual acuity, anterior chamber depth (ACD), and angle width improved significantly in Groups A and B, but not in Group C. The number of antiglaucoma medications used was significantly higher in Group C than in Groups A and B.

**Conclusions:**

Patients who underwent phaco/IOL had better IOP control, improved vision, deeper ACD, and wider angle and required less antiglaucoma medications than those who underwent LPI alone. Performing phaco/IOL weeks to months after the initial LPI did not appear to adversely affect outcomes compared with those of early phaco/IOL.

## 1. Introduction

Primary angle-closure (PAC) is a condition caused by appositional or synechial closure of the anterior chamber angle that leads to aqueous outflow obstruction and intraocular pressure (IOP) elevation. PAC is more common among patients of East Asian origin, with a reported prevalence of 3% among Taiwanese and 1.5% among Guangzhou Chinese aged 50 years or older [1–4]. Acute PAC is an ocular emergency caused by a sudden occlusion of the drainage angle that demands prompt and effective treatment. The conventional treatment for acute PAC includes systemic and topical medications that lower the IOP immediately [5], followed by laser peripheral iridotomy (LPI) or surgical iridotomy to relieve pupillary block, which is considered the most common cause of PAC. However, 38.9%–58.1% of patients who undergo LPI experience chronic increase in IOP or recurrent acute PAC [6–8], indicating a nonpupillary block mechanism. Additionally, it was reported that only 38.1% of Chinese patients with PAC glaucoma (PACG) had pure pupillary block [9]. The nonpupillary block factors include plateau iris syndrome, lens-related factors, and retrolental factors. In the majority of the eyes, more than one mechanism may be involved in the pathogenesis of angle closure [9, 10].

Several studies have suggested that the lens plays a vital role in the pathogenesis of angle closure. A thicker lens may lead to decreased anterior chamber depth (ACD) and angle crowding by pushing the iris periphery against the trabecular meshwork [11–14]. Lens extraction is associated with the deepening of the anterior chamber and widening of the angle [15]. In patients with acute PACG, lens extraction effectively and sustainably reduces IOP and is considered an alternative to the conventional laser treatment [7, 16, 17]. In the Effectiveness in Angle-closure Glaucoma of Lens Extraction (EAGLE) study, clear-lens extraction presented greater efficacy in IOP control and was more cost-effective than conventional LPI; thus, it was suggested as an option for the first-line treatment for patients with PAC or PACG [18].

Although phacoemulsification and intraocular lens implantation (phaco/IOL) performed within days after acute PAC effectively controls IOP and prevents future attack [7, 8], this procedure is not widely accepted as an initial treatment for acute PAC because of surgical difficulty. The current study aimed to compare the effects of performing phaco/IOL early and weeks to months after initial LPI and conventional LPI only on 12 months IOP control as well as changes in visual acuity (VA), spherical equivalent (SE), ACD, angle width, axial length (AL), and number of glaucoma medications used in eyes with acute PAC.

## 2. Methods

The Institutional Review Board of the Chang Gung Memorial Hospital (Taoyuan City, Taiwan) reviewed and approved this study. Written informed consent was obtained from all the participants. The study conformed to the principles of the Declaration of Helsinki.

### 2.1. Participants

The medical records of the patients with medically uncontrolled acute PAC at the Chang Gung Memorial Hospital, Linkou and Keelung branches, between January 2006 and June 2018, were retrospectively reviewed. Patients with eyes presenting typical acute PAC symptoms (ocular or periocular pain, halos, blurred vision, headache, nausea, and vomiting), acutely elevated IOP (>22 mmHg, based on 3 IOP readings), ciliary flush, corneal edema or cloudiness, poorly reactive and partially dilated pupil, glaukomflecken, and gonioscopically occluded drainage angle were included in the study. Patients with secondary glaucoma, prior ocular trauma, or a history of uveitis or other ocular diseases and those who had undergone ocular surgeries previously were excluded from the study. The participants were divided into three groups: Group A, which underwent primary phaco/IOL within 6 weeks after acute PAC; Group B, which underwent LPI initially after acute PAC that was followed by phaco/IOL within 6 months; and Group C, which underwent LPI only.

### 2.2. Initial Medical Treatment

All the participants received an initial medical treatment for acute IOP elevation that included a combination of a topical beta-blocker, alpha-2 adrenergic agonist, carbonic anhydrase inhibitors (CAIs), and additional systemic hyperosmotic agents (intravenous mannitol 1 mg/kg, oral glycerol, or isosorbide 35 mL, twice or thrice a day) or systemic carbonic anhydrase inhibitor (acetazolamide 250 mg four times a day).

### 2.3. Preoperative Evaluations

Patients' demographic and preoperative clinical information, including IOP, best-corrected VA (BCVA), SE, and slit lamp, gonioscopic, and fundus examination findings were reviewed. Contact A-scan biomicroscopy was performed to measure the ACD and AL and to estimate IOL power. VA was evaluated using the Landolt C chart, and the values were converted into the logarithm of the minimum angle of resolution (logMAR). The angle width was graded using the Shaffer classification. The recorded average angle width was the average of Shaffer classification recorded in the superior, inferior, nasal, and temporal quadrants. In Group B, pre-phaco/IOL clinical data were used as the baseline for statistical analysis.

### 2.4. LPI

LPI was performed using a neodymium-doped yttrium aluminum garnet (Nd : YAG) laser after lowering the IOP medically and clearing the cornea. A Wise iridotomy laser lens with coupling gel (Methocel 2%) was applied after inducing topical anesthesia using proparacaine 0.5%. The iridotomy laser lens was placed at the superior periphery of the iris between 10 o'clock and 2 o'clock, preferably at the thinning area. The power setting was 2–3 millijoules (mJ) with a total of 5–15 pulses until iris penetration was achieved. The postoperative treatment included topical prednisolone acetate 1% four times daily tapered over 1 week. The patients were examined after 1 day; 1 week, and 1, 3, 6, 9, and 12 months of LPI.

### 2.5. Surgical Intervention

A single surgeon (WWS) performed phaco/IOL in Groups A and B. The surgical procedure was as follows: after topical anesthesia and sterilization, a standard phacoemulsification procedure was performed through a 2.65 mm clear corneal incision at the 11 o'clock limbus. An additional limbal puncture was performed at the 2 o'clock position for chopper insertion. After injecting viscoelastic material for anterior chamber maintenance, a continuous curvilinear capsulorhexis was performed, followed by hydrodissection. The lens was phacoemulsified, and cortical remnants were removed with irrigation and aspiration. A foldable hydrophobic acrylic IOL (AAB00, AMO, Santa Ana, CA, USA) was implanted in the bag. After complete removal of viscoelastic materials, the clear corneal incision was hydrosealed. After surgery, patients were instructed to use topical tobramycin/dexamethasone combination suspension four times a day. The dosage was rapidly tapered within 1 month of the surgery, with the exact timing depending upon the degree of postoperative inflammation.

### 2.6. Postoperative Assessments

Patients were examined after 1 day, 1 week, and 1, 3, 6, 9, and 12 months of phaco/IOL (Groups A and B) or LPI (Group C) surgery. VA measurement, IOP measurement, slit-lamp examination, and fundus examination were conducted at each visit. Contact A-scan biomicroscopy and gonioscopy were performed at the fourth visit to evaluate changes in ACD, AL, and angle width. For the long-term surgical outcome, complete success was defined as IOP <22 mmHg without antiglaucoma medication; qualified success was defined as IOP <22 mmHg with one or more antiglaucoma medications; and failure was defined as IOP between 22 and 24 mmHg measured on two occasions or IOP ≥24 mmHg on one occasion during the follow-up period.

### 2.7. Statistical Analysis

Continuous variables are presented as mean ± standard deviation. The pre- and postoperative variables in each group were compared using a paired sample *t*-test. Variables among the three groups were compared using one-way analysis of variance with Bonferroni or Fisher's least significant difference post-hoc test. A linear mixed model was constructed to compare the longitudinal IOP changes after treatment among the three groups. The success of IOP control was compared using Fisher's test. The Kaplan–Meier survival curve was plotted for complete success in IOP control among the three groups, and the log rank test was used for verification. All statistical analyses were performed using IBM SPSS Statistics software (version 19, SPSS, Inc., Chicago, IL, USA), and statistical significance was defined as *P* < 0.05.

## 3. Results

Eighty-one eyes with acute PAC were enrolled in the study. Among them, 24 underwent initial phaco/IOL after acute PAC (Group A), 23 underwent LPI initially after acute PAC, followed by phaco/IOL within 6 months (Group B), and 34 underwent LPI alone (Group C). Age, baseline IOP, preoperative VA, vertical C/D (VCD) ratio, and preoperative number of antiglaucoma medications of the patients did not differ significantly among the three groups (Table 1). The mean time intervals between acute PAC attack and phaco/IOL were 20.42 ± 24.50 and 75.39 ± 44.53 days in Groups A and B, respectively. The mean time intervals between acute PAC attack and LPI were 4.83 ± 4.69 and 3.59 ± 4.53 days in Groups B and C, respectively.

After phaco/IOL or laser treatment, the IOP and the number of antiglaucoma medications reduced significantly in all the three groups, although patients in Group C still required more medications than those in Group A or Group B (*P* < 0.001). VA and ACD improved in Groups A and B, but not in Group C. The angle width increased in all the three groups after phaco/IOL or LPI. The Shaffer grading for the anterior chamber angle increased in all the three groups after treatment. No statistically significant difference was noted in postoperative VCD ratio, VA, SE, and AL among the three groups (Table 2).

The results of IOP control 12 months after surgery are presented in Table 3. Complete success and complete plus qualified success (in parentheses) in Groups A, B, and C were 83.33% (95.83%), 78.26% (100%), and 38.23% (88.23%), respectively (Fisher's test *P*=0.001). In Group C, the IOP control failed in 12% patients.

**Figure 1:**
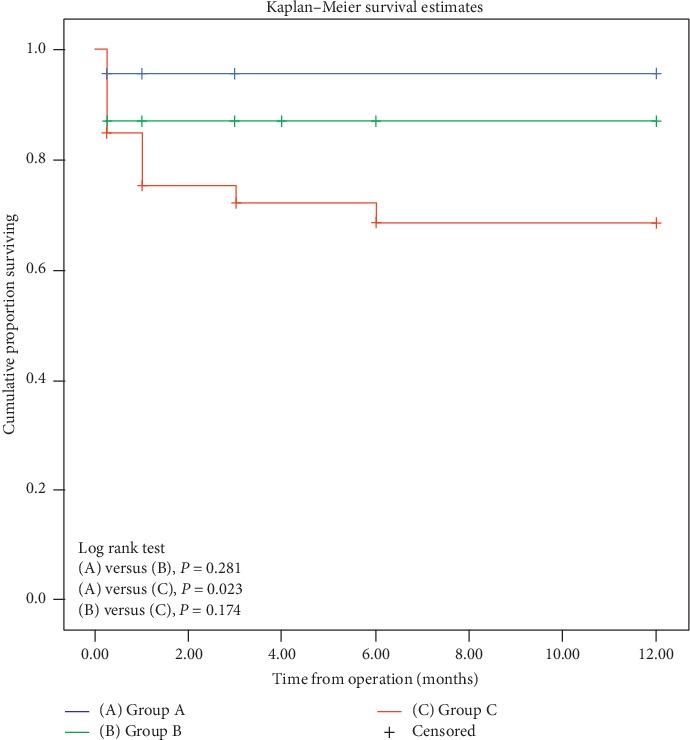
Kaplan–Meier survival curves for complete success after operation.

Figure 1 presents the Kaplan–Meier survival analysis plot for complete success in IOP control. The mean survival times were 11.51 ± 0.48, 10.47 ± 0.83, and 8.67 ± 0.89 months for Groups A, B, and C, respectively. The log rank test demonstrated a statistically significant difference in survival between Groups A and C (*P*=0.023).

**Figure 2:**
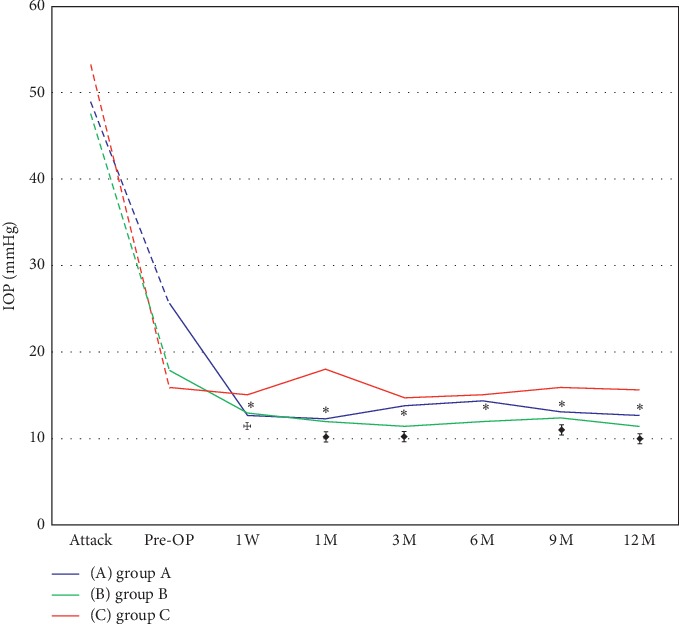
Intraocular pressure changes after operation. Linear mixed model for IOP reduction from baseline (pre-OP) *P* value < 0.05 between (A) and (B)^*☩*^; between (A) and (C)^*∗*^; between (B) and (C)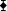. IOP = intraocular pressure, OP = operation, *W* = week, *M* = month(s).

Figure 2 presents the IOP changes after phaco/IOL (Groups A and B) or laser treatment (Group C) in the three groups. The linear mixed model analysis using the preoperative IOP value as baseline revealed that the difference in IOP reduction between Groups A and C was statistically significant at every follow-up point. Significant differences in IOP reduction after 1, 3, 9, and 12 months of surgery were observed between Groups B and C.

## 4. Discussion

The study compared the clinical outcomes in patients with acute PAC who underwent primary phaco/IOL, primary LPI, or postponed phaco/IOL following LPI. It demonstrated that phaco/IOL performed both within weeks and after weeks to months of initial LPI significantly lowered IOP, improved vision, increased ACD and angle width, and reduced the number of antiglaucoma medications in patients with acute PAC.

LPI, the current standard first-line treatment for acute PAC, is the preferred procedure per most of the guidelines [19]. In the current study, although the overall success of long-term IOP control in the LPI only group (Group C) was 88%, it was associated with a higher number of antiglaucoma medications. A previous report on Asian eyes revealed that despite the presence of a patent LPI, 58.1% of eyes with acute PAC developed an increase in IOP on long-term follow-up, with most of them within 6 months, after resolution of the acute attack [6]. LPI relieves pupillary block, a well-established etiology of PACG; however, it was reported that in Chinese patients with PAC, only 38.1% had pure pupillary block [9]. In eyes with acute PAC, although LPI significantly increased the angle width from the baseline, no further increase was observed after 2 weeks [20]. Furthermore, peripheral anterior synechiae (PAS) might progress after LPI. According to Choi and Kim, approximately one-third of the eyes presented PAS progression during a 3-year follow-up period after LPI [21]. On the other hand, the crystalline lens was reported to play a key role in the pathogenesis of PAC, including both pupillary block and nonpupillary block mechanisms [22]. Removing the lens creates more space in the anterior chamber, which is sufficient to achieve IOP control [15]. In our study, patients in Group B who initially received LPI that was followed by phaco/IOL in the next few months were benefited from both procedures, AC deepening in the short term and reduced IOP failure in the long term.

Early phaco/IOL appeared to be more effective in preventing subsequent IOP increase and achieved a lower rate of IOP failure compared to LPI. Lam et al. [7] and Huissan et al. [8] performed phaco/IOL within 1 week after acute PAC attack, and their results were excellent. However, performing phaco within days after acute PAC is technically demanding because of the cloudy cornea, shallow anterior chamber, poor mydriasis, and weakness of the zonular fibers during surgery. In our study, there was no significant difference between Groups A and B in the postoperative IOP control, ocular morphological characteristics, and the number of antiglaucoma medications, indicating that phaco/IOL performed weeks to months after initial LPI did not diminish the effectiveness of phaco in the treatment of acute PAC. Similarly, Römkens et al. compared patients operated within a few days or after a few weeks after acute PAC and found no difference in IOP reduction as well as IOP after 3 months of the surgery [23]. Waiting a few months prior to performing phaco enables doctors to treat acute PAC attacks, reducing IOP, inflammation, and corneal edema and assuring successful surgery.

In the current study, the overall success of long-term IOP control was 95.83% in Group A and 100% in Group B, which is comparable to previous reports [7, 8, 23]. Four eyes (16.66%) in Group A and five eyes (21.73%) in Group B required medical control following phaco/IOL, which was believed to be associated with preexisting PAS that had not been alleviated after surgery. Detailed pre- and postoperative gonioscopic examinations identify the presence and extent of PAS and predict long-term IOP changes. Phaco/IOL combined with goniosynechialysis or endocyclophotocoagulation (ECP) may be considered in eyes with extensive PAS to achieve better IOP control [24–26].

This study had some limitations. This was a retrospective nonrandomized study, with different consequently starting IOP and angle parameters between groups, likely indicating some bias in the treatment selection. Further, owing to small sample size, limited statistical significance was achieved. Finally, the study was specific to Asian eyes, and thus, the findings may not be generalizable to other populations.

## 5. Conclusions

Patients who underwent phaco/IOL had better IOP control, improved vision, deeper ACD, and wider angle and required less antiglaucoma medications than those who underwent LPI alone. Waiting several months to perform phaco/IOL after initial LPI did not appear to adversely affect outcomes when compared with those of early (within days to weeks) phaco/IOL treatment. Clinicians can perform surgeries more confidently after the acute PAC attack subsides, when the cornea is less cloudy and the inflammation is controlled.

## Figures and Tables

**Table 1:** Patient demographics and preoperative ocular characteristics.

	Group A		Group B		Group C		ANOVA *P* value			
	(*n* = 24)		(*n* = 23)		(*n* = 34)		A-B-C^*ε*^	A-B^*Ψ*^	A-C^*Ψ*^	B-C^*Ψ*^
	Mean	SD	Mean	SD	Mean	SD				
Gender M/F	4/20		8/15		11/23					
Laterality OD/OS	14/10		14/9		17/17					
Age (yrs)	70.58	7.38	72.19	7.43	68.14	7.59	0.128			
Attack IOP (mmHg)	48.87	13.17	47.47	14.41	53.17	14.92	0.296			
Pre-OP IOP (mmHg)	25.58	16.24	17.92	10.03	15.86	11.94	0.021	0.141	0.02	1.000
VA (logMAR)	1.08	0.82	0.79	0.51	0.89	0.72	0.347			
VCD ratio	0.51	0.19	0.45	0.23	0.48	0.22	0.671			
No. medication	4.13	1.62	3.74	1.32	4.06	1.14	0.574			

^*ε*^One-way analysis of variance. ^*Ψ*^Bonferroni post-hoc test. M = male, F = female, OD = right eye, OS = left eye, yrs = years old, IOP = intraocular pressure, OP = operation, VA = visual acuity, VCD = vertical cup versus disc, No. = number, and SD = standard deviation.

**Table 2:** Postoperative changes in ocular characteristics and number of antiglaucoma medication.

	Group A					Group B					Group C					Post-OP ocular characteristics			
	(*n* = 24)					(*n* = 23)					(*n* = 34)					ANOVA *P* value			
	Pre-OP		(a) Post-OP			Pre-OP		(b) Post-OP			Pre-OP		(c) Post-OP			(a)-(b)-(c)^†^	(a)-(b)	(a)-(c)	(b)-(c)
	Mean	SD	Mean	SD	*P* value^*∗*^	Mean	SD	Mean	SD	*P* value^*∗*^	Mean	SD	Mean	SD	*P* value^*∗*^				
IOP (mmHg)	25.58	16.24	12.71 (1 week)	5.7	0.001	17.92	10.03	13.01 (1 week)	6.92	0.007	15.86	11.94	15.03 (1 week)	9.12	0.767	0.503			
			12.28 (1 month)	3.01	0.002			11.95 (1 month)	5.48	0.004			18.05 (1 month)	12.96	0.516	0.040	0.909^‡^	0.036^‡^	0.025^‡^
			13.84 (3 months)	6.37	0.057			11.41 (3 months)	3.93	0.004			14.71 (3 months)	4.71	0.657	0.127			
			14.42 (6 months)	5.89	0.168			11.89 (6 months)	4.04	0.092			15.08 (6 months)	6.2	0.777	0.301			
			12.60 (1 year)	3.88	0.146			11.42 (1 year)	3.12	0.047			15.59 (1 year)	6.36	0.92	0.240			
VA (logMAR)	1.08	0.82	0.57	0.43	0.002	0.79	0.51	0.52	0.41	0.002	0.89	0.72	0.70	0.75	0.087	0.481			
SE (D)	0.62	2.96	0.06	1.03	0.815	-0.04	1.92	-0.16	0.60	0.771	0.68	0.55	0.78	2.58	0.942	0.218			
ACD (mm)	2.25	0.36	3.73	0.56	0.001	2.18	0.33	3.41	0.62	<0.001	2.39	0.01	2.35	0.45	0.421	<0.001	0.526^¶^	<0.001^¶^	<0.001^¶^
AL (mm)	22.72	0.96	22.73	1.56	0.426	22.49	0.81	23.01	1.47	0.213	22.31	0.42	22.60	0.43	0.379	0.628			
VCD ratio	0.51	0.19	0.50	0.21	1.000	0.45	0.23	0.48	0.22	0.358	0.48	0.22	0.52	0.23	0.189	0.793			
Shaffer grading^#^	0.00	0.00	3.17	1.20	<0.001	0.00	0.00	3.30	0.97	<0.001	0.00	0.00	2.44	1.21	<0.001	0.011	1.000^¶^	0.060^¶^	0.020^¶^
No. medication	4.13	1.62	0.25	0.68	<0.001	3.74	1.32	0.30	0.77	<0.001	4.06	1.14	1.26	1.42	<0.001	0.001	1.000^¶^	0.002^¶^	0.004^¶^

^*∗*^Paired sample *t*-test. †One-way analysis of variance. ‡Fisher's least significant difference (LSD) post-hoc test. ¶Bonferroni post-hoc test. ^#^The average of Shaffer classification recorded in the superior, inferior, nasal, and temporal quadrants. OP = operation, IOP = intraocular pressure, VA = visual acuity, SE = spherical equivalent, D = diopters, ACD = anterior chamber depth, AL = axial length, VCD = vertical cup versus disc, No. = number, and SD = standard deviation.

**Table 3:** Results of primary outcome in IOP control.

	Group A		Group B		Group C		*p* value
	(*n* = 24)		(*n* = 23)		(*n* = 34)		
	*n*	%	*n*	%	*n*	%	
							0.001^*∗*^
Complete success	20	83.33	18	78.26	13	38.23	
Qualified success	3	12.50	5	21.73	17	50.00	
Failure	1	4.16	0	0.00	4	11.76	

Complete success: IOP <22 mmHg without antiglaucoma medication. Qualified success: IOP <22 mmHg with antiglaucoma medication. Failure: IOP 22–24 mmHg twice or IOP ≥24 mmHg once, one month after intervention.^*∗*^Fisher's test. IOP = intraocular pressure.

## Data Availability

The clinical data used to support the findings of this study are included within the article.
